# A complete 4DCT‐ventilation functional avoidance virtual trial: Developing strategies for prospective clinical trials

**DOI:** 10.1002/acm2.12086

**Published:** 2017-04-24

**Authors:** Timothy Waxweiler, Leah Schubert, Quentin Diot, Austin Faught, Kelly Stuhr, Richard Castillo, Edward Castillo, Thomas Guerrero, Chad Rusthoven, Laurie Gaspar, Brian Kavanagh, Moyed Miften, Yevgeniy Vinogradskiy

**Affiliations:** ^1^ Department of Radiation Oncology University of Colorado School of Medicine Aurora CO USA; ^2^ Department of Radiation Oncology University of Texas Medical Branch Galveston TX USA; ^3^ Department of Radiation Oncology Beaumont Health System Royal Oak MI USA

**Keywords:** CT ventilation, functional imaging, lung cancer

## Abstract

**Introduction:**

4DCT‐ventilation is an exciting new imaging modality that uses 4DCT data to calculate lung‐function maps. Because 4DCTs are acquired as standard of care for lung cancer patients undergoing radiotherapy, 4DCT‐ventiltation provides functional information at no extra dosimetric or monetary cost to the patient. The development of clinical trials is underway to use 4DCT‐ventilation imaging to spare functional lung in patients undergoing radiotherapy. The purpose of this work was to perform a virtual trial using retrospective data to develop the practical aspects of a 4DCT‐ventilation functional avoidance clinical trial.

**Methods:**

The study included 96 stage III lung cancer patients. A 4DCT‐ventilation map was calculated using the patient's 4DCT‐imaging, deformable registration, and a density‐change‐based algorithm. Clinical trial inclusion assessment used quantitative and qualitative metrics based on the patient's spatial ventilation profile. Clinical and functional plans were generated for 25 patients. The functional plan aimed to reduce dose to functional lung while meeting standard target and critical structure constraints. Standard and dose‐function metrics were compared between the clinical and functional plans.

**Results:**

Our data showed that 69% and 59% of stage III patients have regional variability in function based on qualitative and quantitative metrics, respectively. Functional planning demonstrated an average reduction of 2.8 Gy (maximum 8.2 Gy) in the mean dose to functional lung.

**Conclusions:**

Our work demonstrated that 60–70% of stage III patients would be eligible for functional planning and that a typical functional lung mean dose reduction of 2.8 Gy can be expected relative to standard clinical plans. These findings provide salient data for the development of functional clinical trials.

## 1 Introduction

Despite considerable recent technologic advances in thoracic radiotherapy, symptomatic radiation pneumonitis and fibrosis remain serious complications occurring in an estimated 5–50% of patients.^1^ One proposed method for reducing pulmonary toxicity for lung cancer patients is functional avoidance radiotherapy which utilizes functional imaging to selectively spare functional portions of lung in favor of irradiating nonfunctional regions.^2^, ^3^, ^4^, ^5^, ^6^, ^7^, ^8^, ^9^, ^10^, ^11^, ^12^, ^13^ The idea is that sparing functional portions of the lung can reduce the incidence of pulmonary complications.^12^, ^14^ Studies have demonstrated functional avoidance using single‐photon emission computed tomography (SPECT),^2^, ^6^, ^7^, ^8^, ^10^ positron emission tomography (PET),^9^ and magnetic resonance imaging (MRI).^5^

A new lung function imaging technique has been proposed for purposes of functional avoidance^3^, ^4^, ^13^ that calculates ventilation using the patient's 4‐dimensional computed tomography (4DCT) data.^15^, ^16^ Compared with SPECT, PET, and MRI #bib4DCT‐based ventilation (4DCT‐ventilation) offers perhaps the most attractive means toward achieving functional avoidance in radiation oncology because the functional information is obtained using data that is already acquired as standard of care; no additional imaging procedure is necessary. Furthermore, 4DCT‐ventilation does not require a radioactive contrast agent and offers a faster imaging procedure, improved spatial resolution, and an imaging modality that by definition provides anatomical (4DCT) and functional information (4DCT‐ventilation).

Retrospective work has been done to validate 4DCT‐ventilation against nuclear medicine ventilation‐perfusion scans (VQ),^17^, ^18^, ^19^ pulmonary function tests,^17^, ^20^ xenon‐based CT,^16^ MRI,^21^ and PET.^22^ Proof of principle studies have demonstrated the use of 4DCT‐ventilation for functional avoidance,^3^, ^4^, ^13^ and Vinogradskiy et al.^12^ retrospectively showed that doses to 4DCT‐based functional lung better predict for clinical lung toxicity compared with dose alone, suggesting that prospectively incorporating 4DCT‐ventialtion‐based functional information can decrease toxicity.

The retrospective work on 4DCT‐ventilation has paved the way for the development of clinical trials to use 4DCT‐ventilation imaging for functional avoidance radiotherapy at other institutions^23^, ^24^ as well as ours (NCT02528942). Preliminary work on 4DCT‐ventilation‐based functional avoidance has largely focused on demonstrating the feasibility of sparing functional lung; there is little information in the literature that directly addresses the practical aspects of designing a functional avoidance clinical trial. Studies are needed that provide data for guidance on assessment of patient eligibility, planning strategies, and methods of assessment for functional treatment plans. The purpose of this work was, therefore, to perform a complete virtual trial using retrospective data to develop the practical aspects of a 4DCT‐ventilation functional avoidance clinical trial. Specifically, we developed (a) clinical trial inclusion criteria, (b) functional avoidance treatment planning strategies, and (c) characterized the reduction in dose to functional lung.

## 2 Methods and materials

### 2.A Patient population

We retrospectively reviewed 96 locally advanced (stage III) lung cancer patients treated with either 3D conformal or intensity modulated thoracic radiotherapy (IMRT) from MD Anderson or University of Colorado. Patients received a median total dose of 63 Gy (range 45–72 Gy) in 35 fractions (range 15–40 fractions). All patients underwent routine pretreatment 4DCT simulations.

### 2.B 4DCT‐ventilation imaging

**Figure 1:**
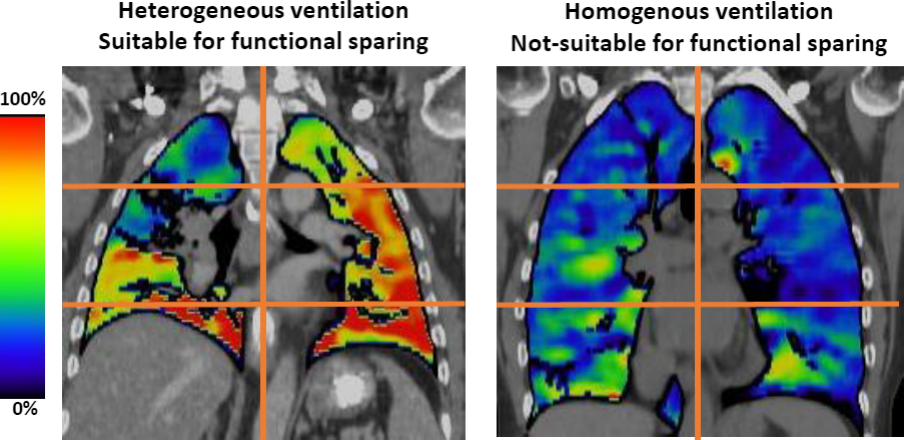
A 4DCT‐ventilation image overlaid with a standard CT is shown for two patients. An example of a patient with homogenous ventilation and a patient with heterogeneous ventilation are shown. The lines demonstrate the concept of dividing the lungs into lung thirds for evaluation.

Each patient's simulation 4DCT data were used to calculate 4DCT‐ventilation maps.^15^, ^25^ The lungs were segmented on the end‐inhale and end‐exhale phases. Lung voxel elements were then mapped from inhale to exhale phase using a deformable image registration (DIR) algorithm with spatial accuracy on the order of 1.25 mm.^26^ The registration was used to apply the HU density‐change equation^15^ to calculate ventilation:

(1) $$\frac{V_{in}-V_{ex}}{V_{ex}}=1000\frac{HU_{in}-HU_{ex}}{HU_{ex}\left(1000+HU_{in}\right)}$$

where *V*_*in*_ and *V*_*ex*_ are the inhale and exhale volumes and *HU*_*in*_ and *HU*_*ex*_ are the inhale and exhale Hounsfield units of the individual lung voxels. Equation (1) calculates the local change in air content for each voxel and produces a 3D map of ventilation function (example shown in Fig. 1). For each 4DCT‐ventilation image, the 4DCT was reviewed for motion artifacts and the DIR was reviewed for discontinuities and errors.

### 2.C Clinical trial inclusion criteria

One critical consideration in determining functional avoidance trial eligibility is a patient's spatial lung function profile. If a patient has homogenous lung function, there is no basis to preferentially spare any regions. Conversely, if a patient's ventilation image is heterogeneous and displays a major ventilation defect, functional avoidance can preferentially deposit dose in the defect area as opposed to the functional region. We previously developed both quantitative and qualitative metrics to assess spatial lung function.^27^ We used the previously developed metrics to aid in the evaluation of trial eligibility of our stage III cohort. The qualitative assessment included a binary metric of whether a ventilation defect was present using consensus from three reviewers. For quantitative analysis, we derived quantitative metrics intended to reflect the degree of heterogeneity of the ventilation image. We computed two metrics using the percent ventilation in each lung third. The percent ventilation in each lung third is a standard metric used in VQ imaging^18^ and is intended to geometrically approximate the ventilation in each lobe (schematically shown in Fig. 1). The two metrics were the percent ventilation in the third containing the tumor (%VTT) and the minimum percent ventilation in the third containing the tumor or any adjacent third (directly superior, inferior, or lateral to the third containing the tumor) [%VTA]. The %VTA metric was intended to detect defects near the primary tumor, and thus most amenable to functional avoidance planning.

In the absence of quantitative guidelines for assessing ventilation heterogeneity, we sought to evaluate how well the quantitative metrics predicted for observer‐identified defects. The assessment was done using logistic regression and receiver operator characteristic (ROC) analysis using the area under the curve (AUC) metric.

### 2.D Functional planning techniques

Based on observer‐identified defects and the %VTA metrics, we determined which patients would be eligible for functional planning (Appendix A). Of the eligible patients, we reduced the subset to patients that were originally treated with IMRT and randomly selected 25 patients for functional planning. The 25 selected patients had a median prescription of 40 Gy (range of 45–70 Gy) with a median target coverage at prescription dose of 94.2% (range 77.9–99.0%). For the 25 selected patients, two plans were created: a functional plan and a standard clinical plan. All organ at risk (OAR) and planning target volume (PTV) contours were taken from the original treatment plan used to treat the patient. The clinical plan was designed following the OAR and PTV guidelines from Radiation Therapy Oncology Group (RTOG) protocol number 0617. RTOG 0617 OAR constraints included a max dose of 50.5 Gy to the spinal cord, volume of total lung getting receiving 20 Gy or more (V20 Gy) less than 37%, esophagus mean less than 34 Gy, and less than 2/3 of the heart volume receiving 45 Gy. The aim was to have 95% cover the planning target volume (PTV) with the prescription dose with a hotspot that could not exceed 120%. In situations where the RTOG 0617 OAR or PTV constraints could not be met, the clinical plan aimed to meet the dosimetry of the original plan used to treat the patient. The reasoning was that the original plan was deemed clinically acceptable at the time of treatment by the clinician and by matching dosimetry parameters, the generated clinical plan could also be considered clinically acceptable.

The functional plan aimed to maximize functional lung sparing while trying to meet RTOG 0617 criteria. A “functional‐avoid” structure was created with auto‐segmentation of functional portions of lung tissue using a threshold of 15%. The 15% threshold was determined using AUC analysis to determine the %VTA value which produced an optimal operative point (largest AUC). The optimal operative point was calculated to be a 15% reduction in ventilation in a given lung third. The 15% reduction was subsequently used to derive the lower limit needed for the auto‐segmentation for the functional‐avoid structure. In other words, the functional‐avoid structure was derived using auto‐segmentation of any lung with no less than a 15% reduction in ventilation. Once auto‐segmentation was applied, the gross or internal target volumes (GTV or ITV) were subsequently subtracted from the functional‐avoid structure.

Both the clinical and functional plans were generated in Eclipse (Varian Medical Systems Inc, Palo Alto, CA, USA) using Volumetric Arc Therapy (VMAT) delivery with the number, direction of arcs, and beam energy at the discretion of the planner. For the functional plan, the reduction in dose to the functional‐avoid structure was accomplished by inserting objective parameters for the functional structure in the optimization and creating favorable arcs directions (by adjusting couch angles, and gantry start and stop angles) that minimized dose to the functional structure. All functional plans were designed to avoid collision and produce deliverable arcs. The arc numbers and directions are presented in the results, and a discussion is provided on the challenges of the functional planning approach.

### 2.E Dose‐function assessment

In the case of functional avoidance radiotherapy, evaluating dose‐volume parameters alone will be insufficient, rather an assessment will be needed that combines both dose and function. We compared the clinical and functional plans using standard dose‐volume and dose‐function metrics. Standard dose metrics included PTV coverage, homogeneity index (HI) (defined as D90%/D5%),^8^ conformity index (CI),^9^ max cord, mean esophagus, mean heart, and mean lung doses (MLD).

Two different types of dose‐function metrics previously presented were calculated:^2^, ^3^, ^4^, ^6^, ^7^, ^10^, ^12^ dose‐function metrics based on the “functional‐avoid” structure and dose‐function metrics based on the entire functional image. Dose‐function metrics based on the structure included mean dose to the structure (MLD) and percentage of structure receiving ≥5 Gy (V5), V10, V20, V30, and V40. Dose‐function metrics based on the entire ventilation image included functionally weighted mean lung dose and functional V5 through V30 based on the dose‐function histogram.^28^ Dose‐function metrics based on the “functional‐avoid” structure are preceded with “S” and metrics based on the entire ventilation image are proceeded with “Im”. Dose‐function metrics were compared between the functional plan and the clinical plans using t‐tests.

## 3 Results

### 3.A Clinical trial inclusion criteria

Of the 96 patients #bib66 (69%) had investigator‐determined clinical ventilation defects. Representative examples of a patient with heterogeneous and homogenous ventilation are shown in Fig. 1. The AUC values evaluating the ability of quantitative 4DCT‐ventilation metrics to predict for investigator‐determined defects ranged from 0.65 to 0.83 with the %VTA metric having the highest AUC (0.83) (Table 1). The %VTA identified 59% of patients as eligible for functional avoidance.

**Table 1:** Area under the curve and logistic regression significance values evaluating the ability of quantitative 4DCT‐ventilation defects to predict for observer‐based defects. Predictive Metrics for Observer‐Based Defects

Metric	AUC	Logistic regression *P* value
IC	0.72	<0.01
CoV	0.65	0.06
%VTT	0.73	<0.01
%VTA	0.83	<0.01

AUC, Area under curve; IC, Ipsilateral to contralateral lung ventilation ratio; CoV, Coefficient of variation; %VTT, Ventilation in the lung third containing the tumor; %VTA, Minimum percent ventilation in the third containing the tumor or any adjacent lung third.

### 3.B Functional planning techniques

To generate the functional plans, a median number of 3 arcs (range 2–4) was utilized. All plans were designed using coplanar arcs except for one case which used a sagittal arc. Twelve (48%) of the plans were generated using directional (ipsilateral) arcs, and 52% used full #bib360° arcs.

### 3.C Dose and dose‐function assessment

Table 2 shows a standard dose metric comparison for the functional plan against the clinical plan. PTV metrics were similar between the clinical and functional plans. The functional plans had worse CI and HI when compared with the clinical plans. The functional plans had reduced lung metrics when compared to the clinical plan, but increased heart, and spinal cord doses.

**Table 2:** A comparison of standard PTV and OAR dose metrics between the clinical plan used to treat the patient and the functional plan. Values shown are averaged among the 25 plans compared. PTV and OAR Dose Metrics Comparison

	Clinical plan	Functional plan	t‐test significance
PTV Metrics			
PTV min (Gy)	48.7	47.7	0.60
PTV max (Gy)	69.6	70.9	<0.01
Conformity Index	1.11	1.15	0.07
Homogeneity Index	0.91	0.88	<0.01
OAR Metrics			
Mean lung dose (Gy)	17.8	16.4	<0.01
Lung V20 (%)	31.0	29.1	0.02
Esophagus mean dose (Gy)	30.1	29.6	0.20
Heart mean dose (Gy)	12.6	13.4	0.45
Cord max dose (Gy)	38.7	42.3	<0.01

PTV, Planning Target Volume; OAR, Organ At Risk.

**Figure 2:**
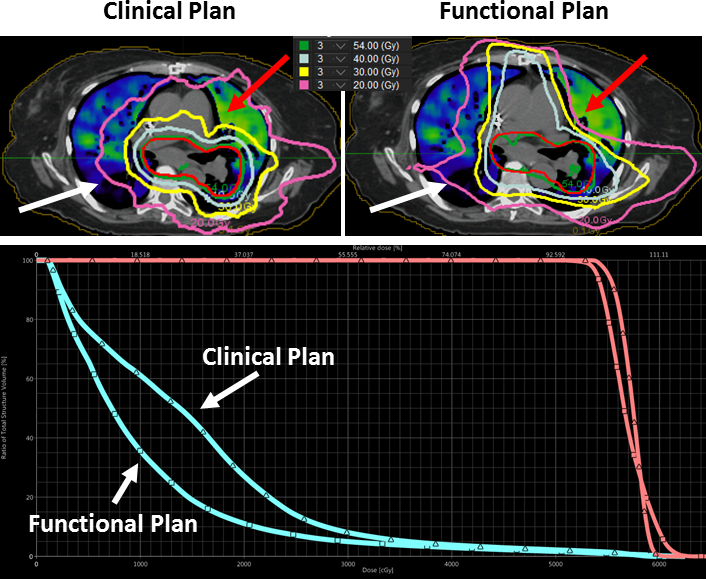
A comparison is provided between a clinical plan and the functional plan. Images on the figure display the planning CT, overlaid functional image, isodose lines, PTV (shown in red), and the DVH curves for the “functional” contour and the PTV. The red arrows highlight where the functional plan was able to spare functional portions of the lung, while the white arrows demonstrate how the functional plan deposited higher doses to nonfunctional lung (when compared with the clinical plan).

**Figure 3:**
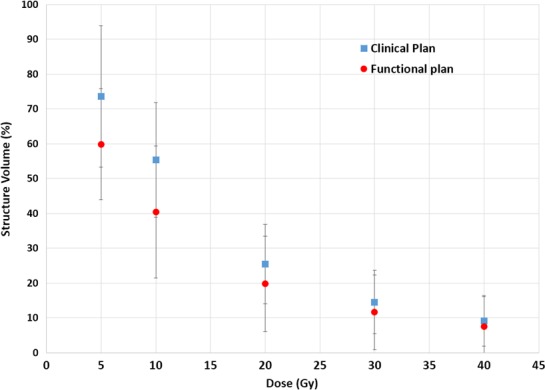
A graphical DVH comparison between the clinical plan (blue squares) and the functional plan (red circles) illustrating the average reduction in dose to the functional contour.

In 24/25 (96%) cases, the dose to functional lung was reduced. A comparison of a functional plan and a clinical plan is shown for a sample patient in Fig. 2. The patient presented in Fig. 2 had an improvement in mean dose to the functional‐avoid structure of 4.6 Gy. Table 3 illustrates that the functional plan reduced structure‐based dose‐function metrics by an average of 2.8 Gy, 13.7%, 14.9%, and 5.6% for the SfMLD, SfV5, SfV10, and SfV20, respectively (all *P* < 0.01) when compared with the clinical plan. Similarly, the image‐based dose‐function metrics were reduced with functional planning (Table 3), but with a smaller magnitude than the structure‐based metrics. The average improvement in structure‐based dose‐function metrics is displayed in a DVH format in Fig. 3.

**Table 3:** A comparison of dose‐function metrics between the clinical plan used to treat the patient and the functional plan. Lung Dose‐Function Metrics Comparison

	Clinical plan	Functional plan	Average difference	Range [Min Max]	*P*
Structure‐based dose‐function metrics					
SfMLD (Gy)	15.6	12.8	−2.8	[−8.2 0.3]	<0.01
SfV5 (%)	73.6	59.9	−13.7	[−53.1 3.3]	<0.01
SfV10 (%)	55.4	40.5	−14.9	[−39.2 1.3]	<0.01
SfV20 (%)	25.4	19.8	−5.6	[−16.0 2.6]	<0.01
SfV30 (%)	14.5	11.7	−2.9	[−9.3 0.2]	<0.01
SfV40 (%)	9.1	7.5	−1.6	[−8.7 1.1]	<0.01
Image‐based dose‐functional metrics					
ImfMLD (Gy)	20.7	18.6	−2.1	[−10.0 12.7]	0.01
ImfV5 (%)	81.4	70.1	−11.3	[−47.0 1.5]	<0.01
ImfV10 (%)	66.9	52.0	−14.9	[−45.7 3.6]	<0.01
ImfV20 (%)	36.9	31.9	−5.0	[−42.1 6.7]	<0.01
ImfV30 (%)	24.2	22.2	−2.0	[−17.2 19.9]	0.12

MLD, mean lung dose, V5, volume receiving greater than 5 Gy, f, functional, S, Structure‐based, Im, Image‐based.

## 4 Discussion

### 4.A Clinical trial inclusion criteria

Our data evaluating spatial lung function suggest that 60–70% of patients have regional function variability conducive to functional avoidance. These results are in line with other studies which report (qualitatively) that approximately 70% of patients exhibit spatially variant ventilation.^6^, ^7^, ^10^ In the current work, we directly applied the previously developed metrics^27^ to a stage III cohort to develop methods to assess whether a patient has enough variability in their spatial lung function to warrant functional avoidance. In the absence of published guidelines for assessing lung function heterogeneity, we believe both the user‐classified defect and the %VTA provide useful information. The user‐identified defect provides a “sanity‐check” and enables the translation of clinical knowledge (from a nuclear medicine radiologist for example) to ventilation clinical trials. The %VTA metric is attractive to use because it provides information about the spatial interplay between the tumor and the ventilation defect. For example, if the patient has a left lower lobe (LLL) mass and a ventilation defect in the right upper lobe (RUL); it is not feasible to design a plan to treat the LLL mass by depositing dose through the ventilation defect in the RUL.

### 4.B Functional planning techniques

The functional planning approach we used was to generate a functional‐avoid structure from the 4DCT‐ventilation image. Some authors proposed a similar structure‐based approach,^2^, ^3^, ^4^, ^5^, ^7^, ^9^, ^13^ while others directly incorporated the 4DCT‐ventilation image into the optimization.^8^, ^10^ Each approach has its pros and cons. A structure‐based approach can be incorporated into any modern planning system, while the image‐based approach may require advanced scripting. Recent 4DCT‐ventilation validation data have shown that the most reliable information from a 4DCT‐ventilation image occurs in ventilation defect regions while there is still some uncertainty in the upper percentiles of function.^17^, ^18^, ^19^, ^21^, ^22^ Converting 4DCT‐ventilation images to structures enables the planner to discard the minor fluctuations in image intensities and focus on the major ventilation features (ventilation defects) of the image. Conversely, directly incorporating the 4DCT‐ventilation image into the optimization algorithm allows for the integration of all imaging information.

All the functional plans were created using rotational IMRT which is the current standard in our clinic and is gaining momentum for thoracic treatments due to its relative simplicity. Standard practice in rotational therapy employs one‐sided partial arcs for lateral disease and full 360° arcs for disease with mostly mediastinal involvement. This treatment paradigm held true for functional planning with the exception of patients that had mediastinal involvement but large ipsilateral ventilation defects; in those instances, one‐sided arcs were dosimetrically beneficial.

### 4.C Dose and dose‐function assessment

Dosimetrically, the functional plans reduced structured‐based mean doses to the functional lung by an average of 2.8 Gy, with reductions of 8.2 Gy possible for individual patients. Evaluating six other studies in the literature produced an average reduction in functional mean lung dose of 2.5 Gy^2^, ^3^, ^4^, ^8^, ^9^, ^13^ with reductions as high as 10 Gy noted. We achieved reductions in SfV5 and SfV20 of 13.7% and 5.6%, respectively. Other studies report fV5 reductions of 10–16%^3^, ^9^, ^13^ and fV20 reductions of 3‐9%.^5^, ^13^, ^29^ Our data are consistent with data from the literature and likely reflect achievable reductions to functional lung in a clinical trial setting. Although final treatment planning guidelines will have to come from future toxicity studies, expected reductions in mean dose, fV20, and fV5 of 2.5 Gy #bib10%, and 5%, respectively, can provide valuable guidance for functional avoidance planning. The idea of the functional clinical trials is that the reduction in mean dose, V5, and V20 to functional lung can reduce the probability of radiation pneumonitis.^1^, ^30^, ^31^, ^32^

As other studies have noted, there are drawbacks to functional plans including an increase in the CI, a reduction in the PTV HI,^4^, ^8^, ^9^ and a potential increase in doses to other OARs, our results echoed the previous studies in that CI and HI got worse with functional planning and dose to other critical thoracic organs (spinal cord, heart, and esophagus) increased. Our observations along with other studies demonstrate that planners should be aware of the possible hotspots, unwanted dose streaks, and increased doses to non‐lung OARs with functional avoidance.

The development of clinical trials is underway to incorporate functional imaging in to thoracic radiotherapy. Our study aims to advance the functional planning field in several key aspects. Prior work has generally presented proof‐of‐principle studies that have performed functional planning for typically 15 patients. We present a complete virtual trial that helps develop practical clinical trial guidelines by comprehensively analyzing trial inclusion for 96 stage III lung cancer patients, discussing the practical aspect of functional planning, and evaluating both structure‐based and image‐based dose‐function dosimetry for 25 patients. Our qualitatively and quantitatively developed metrics suggest that approximately 60–70% of stage III patients would be eligible for functional avoidance based on their spatial lung function profile. We present practical observations of our functional planning experience, including manipulation of the functional‐avoid structure for planning purposes, arc set‐up, and challenges of maintaining dose conformity and heterogeneity. Our functional planning demonstrated that an average reduction of 2.8 Gy to the functional lung can be expected relative to standard thoracic plans. As many clinicians and planners have experience with standard thoracic planning, the 2.8 Gy metric can provide useful guidance to translate the standard thoracic planning knowledge to the functional planning domain. The data provided in the current work on using quantitative and qualitative spatial image assessment for trial eligibility, structure‐based planning strategies, and an average reduction of 2.8 Gy to the functional lung (compared with standard, nonfunctional plans) have all been incorporated into the design of our clinical trial.

We present one way of performing a complete functional avoidance trial. Other strategies for performing various aspect of functional planning have been previously reported.^2^, ^23^, ^24^ Although 4DCT‐ventilation is being used in the prospective clinical trial setting, there are still challenges that add to the uncertainty of the 4DCT‐ventilation calculation process. The quality of the 4DCT‐ventialtion image can degrade with 4DCT imaging artifacts and inaccurate deformable image registration results.^33^ Various methods of normalization are being explored for 4DCT‐ventilation,^34^ and the calculation techniques themselves are still being optimized.^22^ It has been shown that ventilation can improve throughout the course of treatment as the tumor shrinks and the airways open up.^35^ An adaptive approach will be critical for functional planning as the regions of ventilation defect (where higher doses are deposited) can become higher functioning lung as the tumor size decreases throughout treatment. Using the %VTA metric and AUC analysis, we used 15% as the threshold to determine functional lung. Other studies chose thresholds that ranged from 20% to 80%^2^, ^5^, ^13^, ^36^ to identify the functional portions of the lung. This is the first study that we are aware of that chose a threshold for the “functional‐avoid” structure based on a heterogeneity analysis. Final choices of threshold values for generating the “functional contour” and metrics to evaluate ventilation heterogeneity for functional avoidance will be based on clinical experience and clinical outcomes. However, in the absence of clinical outcome data for functional avoidance, we believe the presented ROC analysis provides a strong starting point for determining a threshold for functional lung and ventilation image heterogeneity. The optimal integration of functional planning into radiotherapy will be based on clinical experience, clinical outcomes, and the technology available at any given clinic. However, we believe the data presented in this study provides valuable results for the design of clinical trial strategies.

## 5 Conclusion

Our study presents a virtual trial for functional planning that analyzes trial inclusion for 96 stage III lung cancer patients, discusses the practical aspect of functional avoidance, and evaluates dose‐function dosimetry for 25 patients. Our data show that 59% of patients have regional variability in function that is conducive to functional planning. Our functional planning demonstrated that an average reduction of 2.8 Gy to the mean functional lung dose can be expected relative to standard thoracic plans. The data presented in this study can be used to aid with the design of functional avoidance clinical trials.

## Conflict of Interest

This work was partially funded by grant R01CA200817 (YV, LS, MM, BK, EC, RC, TG) #bib1K01‐CA‐181292‐01 (RC), and State of Colorado grant (YV). The authors have no other relevant conflicts of interest to disclose.

## Appendix 1

In order to assess the spatial ventilation profile for functional avoidance, both quantitative and qualitative metrics were developed. The qualitative assessment included a binary metric of whether or not a ventilation defect was present using consensus from three reviewers. For quantitative analysis, we derived metrics intended to reflect the degree of heterogeneity of the ventilation image. We computed the coefficient of variation (CoV) defined as the ratio of the standard deviation and the mean, the ipsilateral to contralateral (IC) ventilation ratio, and two metrics using the percent ventilation in each lung third. The two metrics were the percent ventilation in the third containing the tumor (%VTT) and the minimum percent ventilation in the third containing the tumor or any adjacent third (directly superior, inferior, or lateral to the third containing the tumor) [%VTA]. The logic behind the %VTA metric is that a ventilation defect may appear downstream of the tumor.

In the absence of quantitative guidelines for assessing ventilation heterogeneity, we sought to evaluate how well the quantitative metrics predicted for observer‐identified defects. The assessment was done using logistic regression, receiver operator characteristic (ROC) analysis, and area under the curve (AUC) evaluation.

We found that the quantitative metric that best predicted for observer‐based defects was the %VTA metrics (percent ventilation in the third containing the tumor or any adjacent third) with an AUC of 0.83 (Table 1). We performed two further analyses using the %VTA metric. The first analysis compared the %VTA metric and user‐identified defects as means to evaluating a patient's heterogeneity profiles. The second analysis used the %VTA metric to help derive a threshold for generating the functional‐avoid structure (presented in the manuscript).

To analyze the relationship between the %VTA metric and user‐identified defects, we calculated standard ROC metrics including the sensitivity, specificity, and overall accuracy (Table A1). It is instructive to compare instances where the %VTA metric and user‐identified defects disagreed. The false positive rate (FPR) in the analysis alludes to instances where the %VTA metric identified a defect but users did not. This scenario can be attributed to situations where the quantitative differences in percent ventilation in each third were more sensitive than what the human observers could decipher. The false negative rate (FNR) refers to situations where the %VTA metric predicted no defect while the users identified a defect. The FNR scenario arose when defects were present but not in an adjacent third relative to the tumor (i.e., further away from the tumor). The distant defects could be due to prior irradiation or chronic obstructive pulmonary disease. In this regard, we believe the %VTA metric provides the more pertinent result for functional avoidance because it is not practical to design treatment plans that place dose through nonventilated lung far away from the tumor. For example, if the patient has a left lower lobe (LLL) mass and a defect in the right upper lobe (RUL), it is not feasible to design a plan to treat the LLL mass by putting dose through the ventilation defect in the RUL.

**Table A1:** Receiver operator characteristic analysis metrics comparing the %VTA metric to user‐identified defects. Analysis of %VTA Metric to Predict User Defined Ventilation Defects

Metrics	Explanation	Value
True positive rate (sensitivity)	% Ventilation said defect and users said defect	0.85
False negative rate (miss)	% Ventilation said no defect but users said defect	0.15
False positive rate (fallout)	% Ventilation said defect but users said no defect	0.47
True negative rate (specificity)	% Ventilation said no defect and users said no defect	0.53
Overall accuracy		0.75
